# Software development for quantitative analysis of brain amyloid PET

**DOI:** 10.1002/brb3.2499

**Published:** 2022-02-08

**Authors:** Hiroshi Matsuda, Tensho Yamao

**Affiliations:** ^1^ Department of Biofunctional Imaging Fukushima Medical University Fukushima City Fukushima Japan; ^2^ Drug Discovery and Cyclotron Research Center Southern Tohoku Research Institute for Neuroscience Koriyama Fukushima Japan; ^3^ Department of Radiology National Center of Neurology and Psychiatry Kodaira Tokyo Japan; ^4^ Department of Radiological Sciences School of Health Sciences Fukushima Medical University Sakae Fukushima Japan

**Keywords:** amyloid, brain, Centiloid scale, PET, quantification

## Abstract

**Introduction:**

Centiloid (CL) scaling has become a standard quantitative measure in amyloid PET because it allows the direct comparison of results across sites, even when different analytical methods or PET tracers are used.

**Methods:**

In the present study, we developed new standalone software to easily handle a pipeline for accurate calculation of the CL scale for the five currently available amyloid PET tracers—^11^C‐PiB, ^18^F‐florbetapir, ^18^F‐flutemetamol, ^18^F‐florbetaben, and ^18^F‐NAV4694. This pipeline requires reorientation and coregistration of PET and MRI, anatomic standardization of coregistered PET to a standardized space using a warping parameter for coregistered MRI, application of standard volumes of interest (VOIs) to the warped PET, calculation of the standardized uptake value ratio (SUVR) for the target VOIs, and finally conversion of the SUVR to the CL scale. The PET data for these tracers were collected from the publicly available Global Alzheimer's Association Interactive Network (GAAIN) repository. We also developed software to map Z‐scores for the statistical comparison of a patient's PET data with a negative control database obtained from young healthy controls in the GAAIN repository.

**Results:**

When whole cerebellum or whole cerebellum plus brainstem was chosen as the reference area, an excellent correlation was found between the CL scale calculated by this software and the CL scale published by GAAIN. There were no significant differences in the detection performance of significant amyloid accumulation using Z‐score mapping between each ^18^F‐labeled tracer and ^11^C‐PiB. The cutoff CL values providing the most accurate detection of regional amyloid positivity in Z‐score mapping were 11.8, 14.4, 14.7, 15.6, and 17.7 in the posterior cingulate gyrus and precuneus, frontal cortex, temporal cortex, parietal cortex, and striatum, respectively.

**Conclusion:**

This software is able to not only provide reliable calculation of the global CL scale but also detect significant local amyloid accumulation in an individual patient.

## Graphical abstract

Centiloid scale calculation

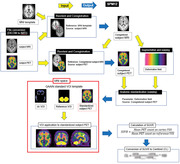

## 1 INTRODUCTION

Amyloid positron emission tomography (PET) increases the diagnostic accuracy of Alzheimer's disease (AD) and non‐AD. Because the binary classification of positive and negative amyloid PET findings is routinely based on visual interpretation, equivocation is inevitable and leads to interrater variability (Hosokawa et al., 2015). Equivocal findings should be avoided when the indication is being determined for the disease‐modifying drugs currently under development. Accordingly, quantitative analysis has been proposed as an aid to visual interpretation (Collij et al., 2019; Matsuda et al., 2021).

The standardized uptake value ratio (SUVR) has been widely applied to the quantitative analysis of amyloid PET. However, SUVR values depend not only on the target and reference regions used, but also on the particular amyloid PET tracer. This variability can be resolved through a Centiloid (CL) scaling process that standardizes the quantitative amyloid imaging measures by standardizing the outcome of each analytical method or PET ligand to a scale from 0 to 100 (Klunk et al., 2015). The CL scale offers a direct comparison of results across institutions, even when different analytical methods or tracers are used, and may enable the clear definition of cutoffs for amyloid positivity. To determine the CL scale, it is necessary to follow the method put forward by the Global Alzheimer's Association Interactive Network (GAAIN, http://www.gaain.org/centiloid‐project). However, this approach is time‐consuming because it requires numerous steps to process the PET images and the corresponding MRI data and the use of multiple software packages. Furthermore, to more reliably determine amyloid positivity, the local pattern of amyloid accumulation should be captured, in addition to the global CL scale. To resolve these issues, we have developed standalone software for both calculating the global CL scale and detecting which brain regions have statistically significant amyloid accumulation. In the present study, we describe the details of this new software and its validation.

## 2 MATERIALS AND METHODS

### 2.1 Data availability

This study was conducted using datasets collected from the publicly available GAAIN repository. These datasets comprised 495 PET images obtained using five different amyloid PET tracers (^11^C‐PiB, ^18^F‐florbetapir, ^18^F‐flutemetamol, ^18^F‐florbetaben, and ^18^F‐NAV4694) and the corresponding three‐dimensional T1‐weighted MRI data of patients with AD, frontotemporal dementia, and mild cognitive impairment and of young and elderly healthy controls (Table 1). Acquisition of PET scan images were done from 50 to 70 min for ^11^C‐PiB and ^18^F‐NAV4694, from 50 to 60 min for ^18^F‐florbetapir, and 90 to 110 min for ^18^F‐flutemetamol and ^18^F‐florbetaben after administration of the PET tracer.

**TABLE 1:** Datasets of amyloid PET tracers, MRI, and subjects in GAAIN repository

		Subjects			
PET tracer	MRI	Total number	Patients ( age)	EHC (age)	YHC (age)
^11^C‐PiB	3D T1WI	79	45 AD (N/A)	0 (N/A)	34(N/A)
^18^F‐florbetapir + ^11^C‐PiB	3D T1WI	46	17 AD (51–76 yo), 7 MCI (64–89 yo), 3 At risk elderly (78–83 yo)	6 (51–75 yo)	13 (21–35 yo)
^18^F‐flutemetamol + ^11^C‐PiB	3D T1WI	73	20 AD(60–81 yo), 20 MCI (57–83 yo)	9 (47–75 yo)	24 (31–45 yo)
^18^F‐florbetaben + ^11^C‐PiB	3D T1WI	35	8 AD (61–77 yo), 9 MCI (65–80 yo), 2 FTD (68–79 yo)	6 (63–84 yo)	10 (25–46 yo)
^18^F‐NAV4694 + ^11^C‐PiB	3D T1WI	54	7 AD (59–83 yo), 9 MCI (60–89 yo), 3 FTD (63–72 yo)	25 (57–89 yo)	10 (26–45 yo)

AD, Alzheimer's disease; MCI, mild cognitive impairment; FTD, frontotemporal dementia; EHC, elderly healthy control; YHC, young healthy control; 3D T1WI, three‐dimensional T1‐weighted image; N/A, not available; yo, years old.

### 2.2 Processing pipeline of the software

The software developed in this study comprises two distinct processes: calculation of the CL scale from each subject's amyloid PET and MRI, and a statistical comparison of each subject's amyloid PET with a database of negative amyloid PET results obtained from young healthy controls.

**FIGURE 1:**
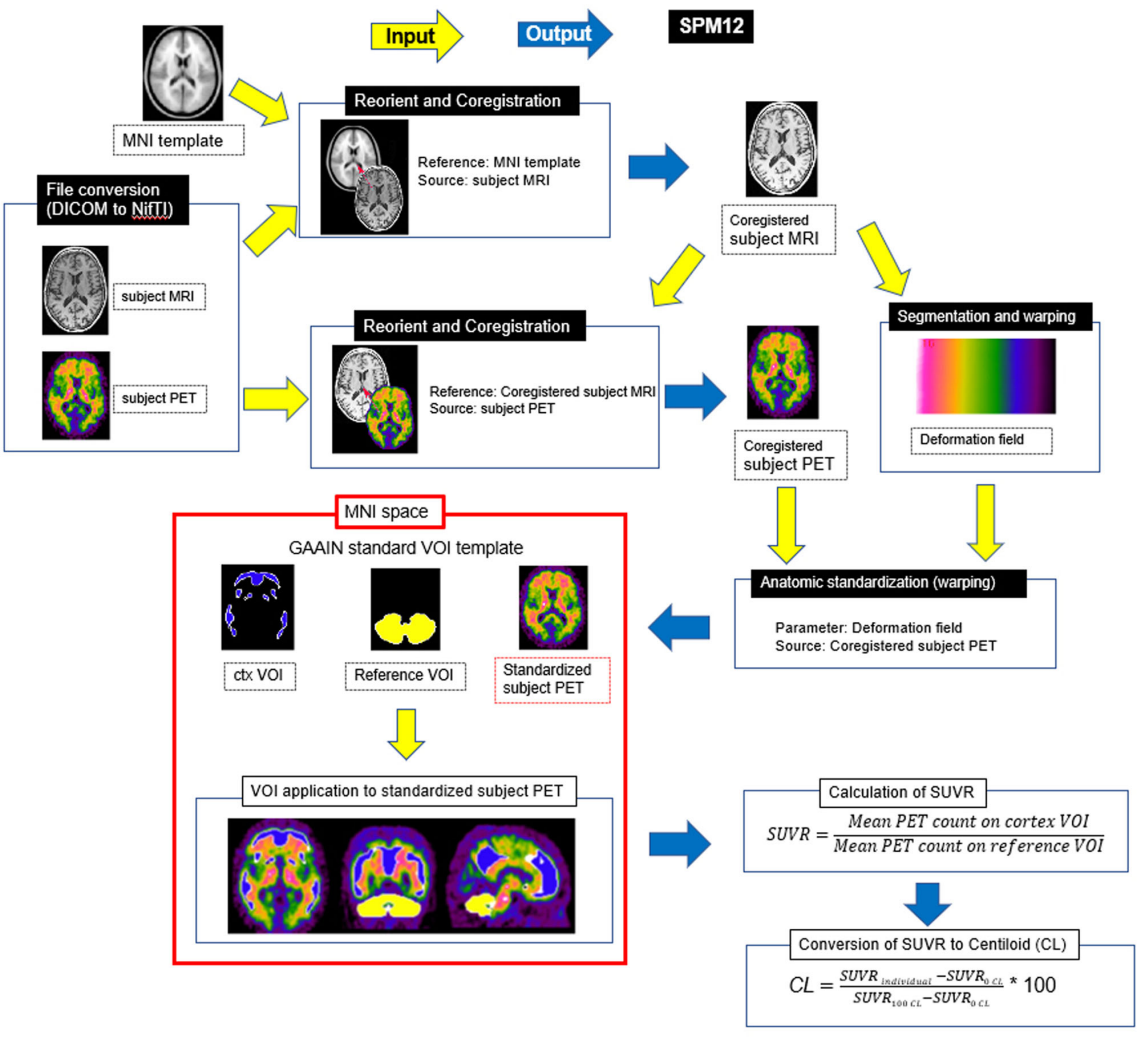
Processing pipeline for quantitative measurements of amyloid accumulation in the target area of the cerebral cortex and striatum. The subject MRI was reoriented by setting the origin around the anterior commissure and coregistered to the Montreal Neurological Institute (MNI) template (avg152T1.nii). The subject PET was then reoriented also by setting the origin around the anterior commissure and coregistered to the coregistered subject MRI. Then, the coregistered subject MRI was warped into MNI space using unified segmentation. The parameters of the deformation field in this warping are applied to the coregistered subject PET images for anatomic standardization into MNI space. These translations were performed using the Statistical Parametric Mapping (SPM) 12 software. The SUVR is calculated from the amyloid PET counts in the cerebral cortical and striatal (ctx) volumes of interest (VOIs) and in a reference VOI using Global Alzheimer's Association Initiative Network (GAAIN) standard VOI templates. Next, the SUVR is converted to CL values using a direct conversion equation. Processing with a black background was SPM12

**FIGURE 2:**
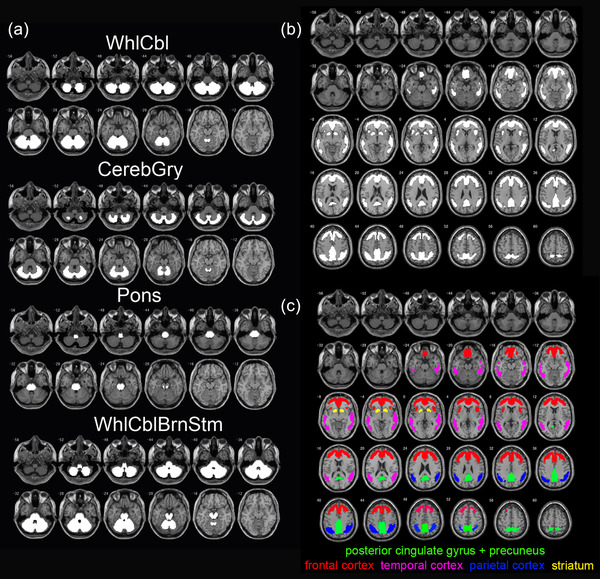
Standard VOI templates. (a) Reference VOIs. The GAAIN template for reference VOIs is represented by white areas. WhlCbl, whole cerebellum; CerebGry, cerebellar gray matter; WhlCblBrnStm, whole cerebellum plus brainstem. (b) Target cortical and striatal VOIs. The GAAIN template for target cortical and striatal VOI is represented by white areas. (c) Division of the target cortical and striatal areas into five regions. Posterior cingulate gyrus plus precuneus (green), frontal cortex (red), temporal cortex (pink), parietal cortex (blue), and striatum (yellow)

The first process for quantitative analysis using the SUVR and a 100‐point scale termed the CL scale is illustrated in Figure 1. First, a pair of subject PET and subject MRI images is input in DICOM or NIfTI format. Then, the subject MRI was reoriented by setting the origin around the anterior commissure and coregistered to the Montreal Neurological Institute (MNI) template (avg152T1.nii). The subject PET was then reoriented also by setting the origin around the anterior commissure and coregistered to the coregistered subject MRI. Then, the coregistered subject MRI was warped into MNI space using unified segmentation (Ashburner & Friston, 2005). The parameters of the deformation field in this warping are applied to the coregistered subject PET images for anatomic standardization into MNI space. These translations were performed using the Statistical Parametric Mapping (SPM) 12 software (https://www.fil.ion.ucl.ac.uk/spm). The SUVR is calculated from standardized subject PET counts in four reference regions—the whole cerebellum (GAAIN WhlCbl VOI), cerebellar gray matter (GAAIN CerebGry VOI), pons (GAAIN Pons VOI), and whole cerebellum plus brainstem (GAAIN WhlCblBrnStm VOI) (Figure 2a)—and in the global cortical target region (GAAIN ctx VOI) (Figure 2b). Finally, the SUVR is converted to CL values using direct conversion equations (Table 2) for each PET tracer, as described in previous reports (Battle et al., 2018; Klunk et al., 2015; Navitsky et al., 2018; Rowe et al., 2016; Rowe et al., 2017).

**TABLE 2:** Conversion equations from SUVR to CL scale

	Amyloid PET tracer				
Reference VOI	^11^C‐PiB	^18^F‐florbetapir	^18^F‐flutemetamol	^18^F‐florbetaben	^18^F‐NAV4694
WhlCbl	–94.64+93.75 × SUVR	–182.23+175.17 × SUVR	–121.16+121.42 × SUVR	–155.06+153.53 × SUVR	–87.99+85.34 × SUVR
CerebGry	–93.06+79.52 × SUVR	N/A	N/A	–152.93+128.95 × SUVR	–86.21+71.70 × SUVR
WhlCblBmStm	–98.42+129.28 × SUVR	N/A	N/A	–156.65+217.92 × SUVR	–93.44+122.27 × SUVR
Pons	–95.58+99.68 × SUVR	N/A	N/A	–155.63+163.27 × SUVR	–89.39+91.42 × SUVR

WhlCbl, whole cerebellum; CerebGry, cerebellar graymatter; WhlCblBrnStm, whole cerebellum plus brain stem; N/A, not available.

**FIGURE 3:**
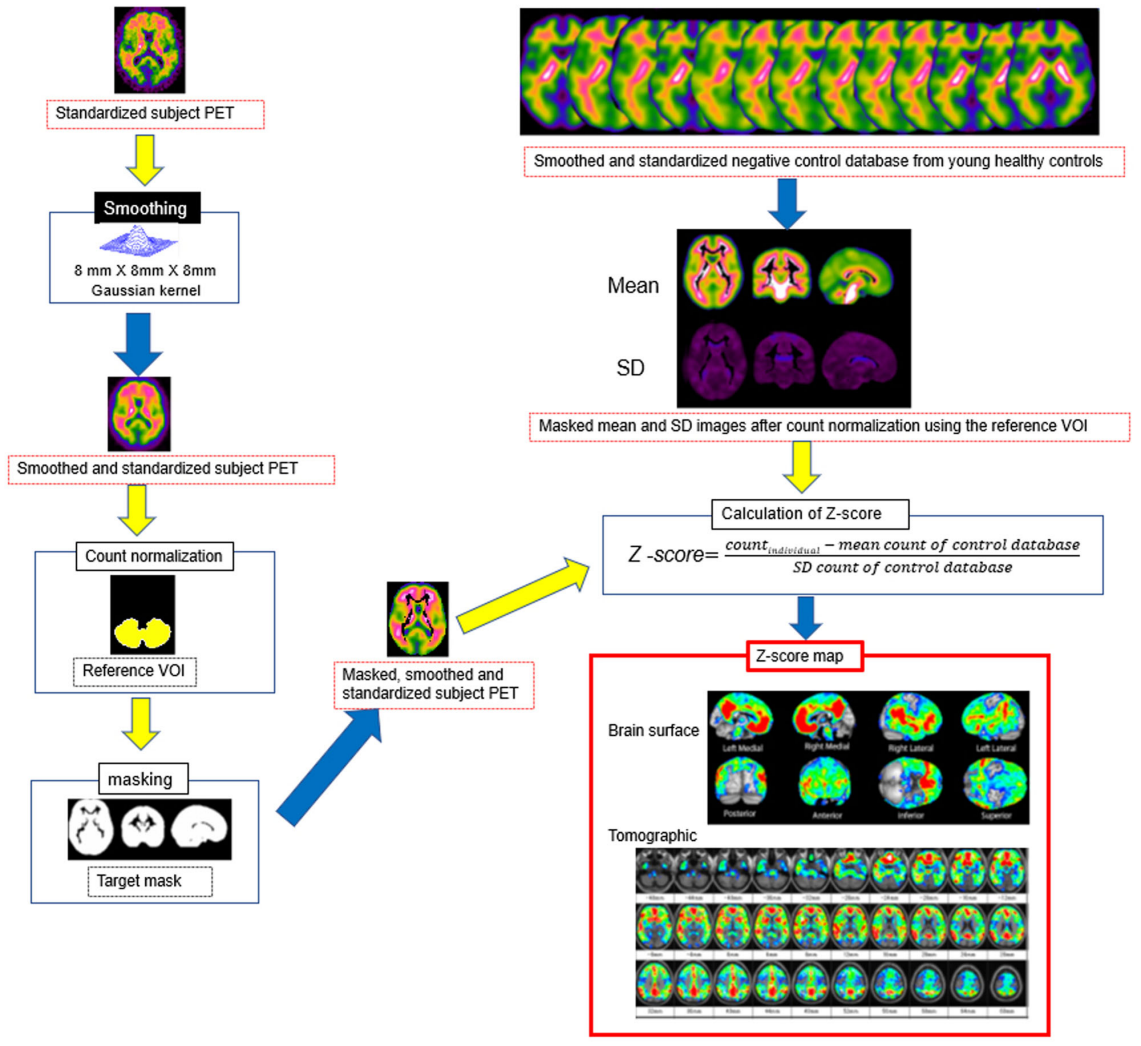
Processing pipeline for comparison of each subject's PET data with a negative normal database comprising young healthy controls. The standardized subject PET is smoothed using an 8‐mm^3^ Gaussian kernel. The smoothed and standardized subject PET images are then masked to remove white matter areas with high counts after normalization of the PET count using the reference VOI count. A Z‐score is calculated from a comparison of masked, smoothed, and standardized subject PET images with masked mean and standard deviation PET images generated from an amyloid‐negative control database comprising smoothed and standardized subject PET images of young healthy controls from the GAAIN dataset repository. A Z‐score map is displayed by overlay on tomographic sections with a contour of the target cortical and striatal area and surface rendering of the standardized brain MRI

**FIGURE 4:**
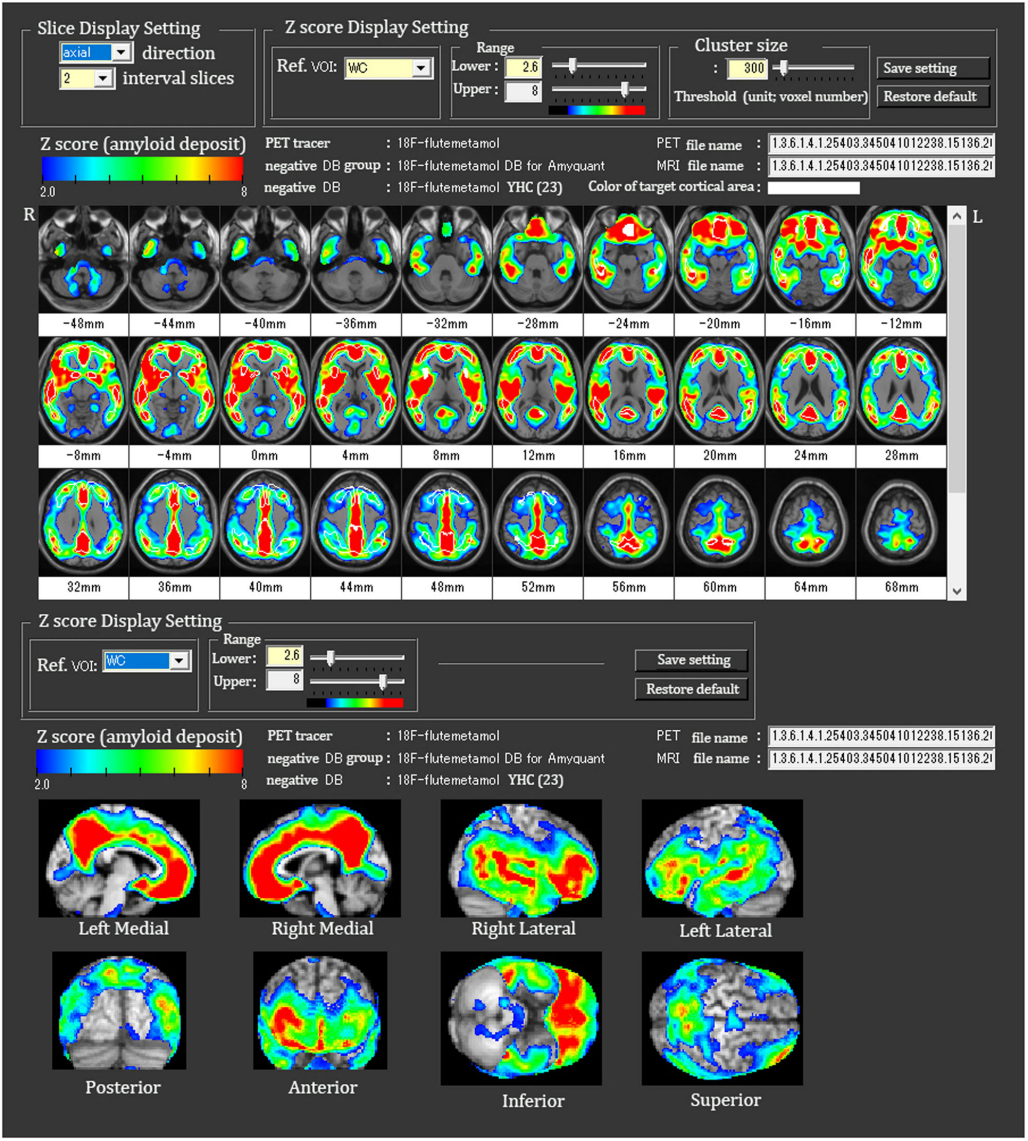
Z‐score mapping on tomographic sections and surface rendering of the standardized brain MRI. The target cortical and striatal areas are contoured by white lines. The lower and upper levels of the Z‐score can be changed along with a threshold of a cluster size

The second process, which involves comparison of each subject's PET data with a negative normal database comprising young healthy controls, is illustrated in Figure 3. First, the standardized subject PET images are smoothed using an 8‐mm^3^ Gaussian kernel. The smoothed and standardized subject PET images are then masked to remove white matter areas with high counts after normalization of the PET count using a reference volume of interest (VOI) count. Masked mean and standard deviation PET images are generated from an amyloid‐negative control database comprising smoothed and standardized subject PET images of young healthy controls with a CL score less than 10 published on the GAAIN website for each PET tracer (34 subjects for ^11^C‐PiB, 10 for ^18^F‐florbetapir, 23 for ^18^F‐flutemetamol, 10 for ^18^F‐florbetaben, and 10 for ^18^F‐NAV4694). A CL score less than 10 has been reported to be optimal for excluding neuritic plaques in comparisons of amyloid PET measures with neuropathological findings (Amadoru et al., 2020). A Z‐score map for masked, smoothed, and standardized subject PET data is displayed by overlay on tomographic sections with a contour of the target cortical and striatal areas and with surface rendering (Figure 4) of the standardized brain MRI data using the following equation: Z‐score = ([individual count] – [mean count of control database])/(standard deviation count of control database). In the Z‐score mapping display, we can change the upper and lower Z‐score levels and the cluster size threshold.

After reorientation of the PET and MRI images using SPM12, these two processes run automatically and sequentially in standalone software on a Windows operating system. The software, named “Amyquant,” requires about 5 min to complete all of the steps for a single subject using a 64‐bit laptop (CPU, Intel^®^ Core™ i7, 1.90 GHz; memory, 16 GB).

### 2.3 Validation of the software for CL calculation

The CL scales calculated using the present software were compared with the CL scales published on the GAAIN website for each PET tracer and each reference VOI. For validation, as defined by Klunk et al. (2015), the slope should be between 0.98 and 1.02 and the intercept between −2 and +2 CL for a linear regression equation and the *R* ^2^ correlation coefficient should exceed 0.98.

### 2.4 Evaluation of Z‐score mapping

To evaluate the regional detectability of significant amyloid accumulation using each ^18^F‐labeled tracer, we compared the presence or absence of areas with significant amyloid accumulation in five regions (the posterior cingulate cortex and precuneus, frontal cortex, temporal cortex, parietal cortex, and striatum; Figure 2c) of the target cortical areas between each ^18^F‐labeled tracer and the corresponding ^11^C‐PiB PET images from the same individuals (χ^2^ test). To avoid false positives, we set a Z‐score threshold of 2.6 corresponding to *p* < 0.01 with sufficiently large cluster size of 300 voxels (2.4 cc). We then performed receiver operating characteristic (ROC) analysis on the pooled data of all PET tracers to evaluate the relationship between the global CL scale and the positive or negative findings for regional amyloid accumulation.

## 3 RESULTS

When the whole cerebellum or whole cerebellum plus brainstem was chosen as the reference VOI, an excellent correlation between the CL scales calculated by this software and those published on the GAAIN website was confirmed by the fact that the correlation coefficient, as well as the slope and intercept of the linear regression equation, were within the range allowed by Klunk et al. (2015) in each of the four chosen reference regions using the five different PET tracers (Table 3). When cerebellar gray matter was chosen as the reference VOI, the intercept for ^18^F‐florbetaben exceeded the allowed range. When the pons was chosen as the reference VOI, the slope for ^18^F‐florbetaben and ^18^F‐NAV4694 and the correlation coefficient for ^18^F‐florbetaben exceeded the allowed ranges.

**TABLE 3:** Correlation coefficient and linear regression equation between the CL scales calculated by the present software and those published on the GAAIN website

		Reference VOI											
		WhlCbl			CerebGry			WhlCblBrnStm			Pons		
			Linear regression equation			Linear regression equation			Linear regression equation			Linear regression equation	
Amyloid PET tracer	Number of subjects	*R* ^2^	Slope	Intercept	*R* ^2^	Slope	Intercept	*R* ^2^	Slope	Intercept	*R* ^2^	Slope	Intercept
^11^C‐PiB	287	0.993	1.01	0.64	0.993	1.01	1.36	0.996	1.00	0.27	0.996	0.98	−1.64
^18^F‐florbetapir	46	0.991	1.00	0.1	N/A	N/A	N/A	N/A	N/A	N/A	N/A	N/A	N/A
^18^F‐flutemetamol	73	0.982	1.01	0.27	N/A	N/A	N/A	N/A	N/A	N/A	N/A	N/A	N/A
^18^F‐florbetaben	35	0.997	0.99	0.97	0.994	0.98	2.6	0.997	0.99	0.17	0.942	0.94	1.5
^18^F‐NAV4694	54	0.998	0.99	0.02	0.997	1.00	0.6	0.998	0.99	−0.29	0.997	0.97	−1.67

VOI, volume of interest; WhlCbl, whole cerebellum; CerebGry, cerebellar graymatter; WhlCblBrnStm, whole cerebellum plus brain stem; N/A, not available.

A comparison of the detection performance of significant amyloid accumulation in the five regions using Z‐score mapping revealed no statistically significant difference (*p* > 0.3) between each ^18^F‐labeled tracer and the corresponding ^11^C‐PiB in the same individuals (Table 4). In the 495 amyloid PET studies, ROC analysis between Z‐score mapping and the global CL scale showed that the CL scale could determine the positivity of local amyloid accumulation with high accuracy (Table 5). The cutoff CL values for amyloid positivity were 11.8, 14.4, 14.7, 15.6, and 17.7 in the posterior cingulate gyrus and precuneus, frontal cortex, temporal cortex, parietal cortex, and striatum, respectively.

**TABLE 4:** Comparison of negative or positive findings in target regions using Z‐score mapping between 18F‐labeled tracer and 11C‐PiB

	Target region									
	Posterior cingulate gyrus/precuneus		Frontal cortex		Temporal cortex		Parietal cortex		Striatum	
Combination of amyloid PET tracers	Negative	Positive	Negative	Positive	Negative	Positive	Negative	Positive	Negative	Positive
^18^F‐florbetapir	20	26	23	23	24	22	24	22	27	19
^11^C‐PiB	23	23	24	22	24	22	25	21	25	21
χ2	0.393		0.044		0		0.044		0.177	
*p* Value	0.531		0.835		1		0.835		0.674	
^18^F‐flutemetamol	43	30	42	31	43	30	43	30	43	30
^11^C‐PiB	42	31	43	30	43	30	43	30	43	30
χ2	0.028		0.028		0		0		0	
*p* Value	0.867		0.867		1		1		1	
^18^F‐florbetaben	15	20	13	22	17	18	18	17	18	17
^11^C‐PiB	17	18	16	19	17	18	17	18	18	17
χ^2^	0.543		0.238		0.238		0.057		0	
*p* Value	0.461		0.625		0.625		0.811		1	
^18^F‐NAV4694	29	25	36	18	35	19	36	18	39	15
^11^C‐PiB	33	21	34	20	37	17	38	16	39	15
χ2	0.607		0.162		0.167		0.172		0	
*p* Value	0.436		0.687		0.683		0.678		1	

**TABLE 5:** ROC analysis between regional positivity using Z‐score mapping and the global CL scale for the pooled data of all PET tracers

	Target region				
	Posterior cingulate gyrus/precuneus	Frontal cortex	Temporal cortex	Parietal cortex	Striatum
Area under curve	0.983	0.993	0.991	0.999	0.999
Sensitivity	92.4	94.3	96.3	98.6	99.5
Specificity	98.8	100	99.2	99.6	98.2
Accuracy	95.7	97.3	97.9	99.2	98.8
Cut‐off CL value	11.8	14.4	14.7	15.6	17.7

## 4 DISCUSSION

In the present study, we developed standalone software for quantifying amyloid PET. This software provides both the global level of amyloid accumulation in the cerebral cortex and striatum as CL values and a Z‐score map displaying local amyloid accumulation with statistical significance versus a negative database generated from young controls. The accuracy of the CL values calculated using the present software was validated by comparison with the values published on the GAAIN website. Z‐score mapping was able to elucidate the exact location of significant increase of amyloid accumulation. As the CL score increased, the local amyloid accumulation became significant in the order of the posterior cingulate gyrus and precuneus, frontal cortex, temporal cortex, parietal cortex, and striatum.

Selection of the whole cerebellum or whole cerebellum plus brainstem as the reference area revealed an excellent correlation between the CL values calculated by the present software and those published on the GAAIN website, irrespective of the PET tracers used. On the other hand, selection of the cerebellar gray matter resulted in a worse correlation in ^18^F‐labeled tracers, and selection of the pons resulted in the worst correlation. These results are compatible with those of a previous report by Klunk et al. (2015). They reported the SUVR values of ^11^C‐PiB PET in AD patients and young healthy controls using the same standard reference regions after anatomic standardization of PET images using the same manner as in the present study. They demonstrated smaller variance in the SUVR in each group and a larger effect size with the whole cerebellum or whole cerebellum plus brainstem than with the cerebellar gray matter or pons. The pons had the largest variance and smallest effect size. They speculated that this poor performance with cerebellar gray matter may result from difficulty in removing the adjacent cerebellar white matter with high accumulation and that the worst performance with the pons may result from less accurate anatomic standardization in SPM for brainstem structures compared with cortical structures.

Z‐score analysis to compare the subject's PET data with a negative database of young controls demonstrated equivalent sensitivity of statistically significant regional amyloid accumulation between each ^18^F‐labeled tracer and ^11^C‐PiB. In this cross‐sectional study, using the global CL scale as an indicator of AD progression, amyloid accumulation began in the posterior cingulate gyrus and precuneus and spread to the frontal cortex, lateral temporal cortex, and parietal cortex and finally to the striatum. Furthermore, the global CL scale can be used to estimate the graded positivity of local amyloid accumulation with high accuracy. Several spatial and temporal orderings of amyloid positivity using PET have been reported, and the results are similar to those of the present study. Huang et al. (2013) found early amyloid accumulation in the precuneus, frontal cortex, and posterior cingulate gyrus and later deposition in the parietal, occipital, and lateral temporal cortex. Other studies (Cho et al., 2016; Mattsson et al., 2015; Palmqvist et al., 2017; Villeneuve et al., 2015) have reported a similar order of amyloid positivity in cortical regions, with early deposition in the medial frontal cortex and precuneus and posterior cingulate cortex followed by accumulation in the lateral temporal cortex and parietal cortex. On the other hand, it has been reported that striatal deposition follows cortical deposition (Hanseeuw et al., 2018). The current cutoff CL value of 11.8 for amyloid positivity in the posterior cingulate gyrus and precuneus is in good agreement with the CL threshold of 12.2 associated with the Consortium to Establish a Registry for Alzheimer's Disease (CERAD) moderate‐to‐frequent neuritic plaques in the comparative study between standard postmortem measures of AD neuropathology and antemortem ^11^C‐PiB PET (La Joie et al., 2019)

This study has some limitations. First, our newly developed software is not fully automatic. The first step requires setting of the origin of the PET and MRI images around the anterior commissure to avoid inaccurate coregistration due to the large distance between the PET and MRI origins. Semiautomatic or automatic reorientation would be preferable. Second, the small number of young control subjects from the amyloid‐negative GAAIN database comprising datasets for ^18^F‐labeled tracers could cause false‐positive or ‐negative findings in Z‐score analysis. Although there were no significant differences in the detection performance of regional positivity between ^18^F‐labeled tracers and ^11^C‐PiB, a larger negative database may be necessary to increase the accuracy of Z‐score analysis for ^18^F‐labeled tracers. Third, a longitudinal study in the same individuals may be necessary for more accurate comprehension of the spatial and temporal ordering of amyloid pathology in AD.

## 5 CONCLUSIONS

We developed standalone quantitative software for amyloid PET. In addition to reliably calculating the global CL scale, this software can detect significant local amyloid accumulation in an individual patient by comparison with a negative database of young healthy controls. This software can be applied to the five currently available amyloid PET tracers.

## CONFLICT OF INTEREST

No authors had conflicts of interest relevant to this study.

## AUTHOR CONTRIBUTIONS

H. Matsuda and T. Yamao developed the study concept and contributed to the study design. H. Matsuda performed the data analysis and interpretation. All authors provide revisions and approved the final version of the paper for submission.

### PEER REVIEW

The peer review history for this article is available at https://publons.com/publon/10.1002/brb3.2499.

## References

[brb32499-bib-0001] Amadoru, S. , Doré, V. , McLean, C. A. , Hinton, F. , Shepherd, C. E. , Halliday, G. M. , Leyton, C. E. , Yates, P. A. , Hodges, J. R. , Masters, C. L. , Villemagne, V. L. , & Rowe, C. C. (2020). Comparison of amyloid PET measured in Centiloid units with neuropathological findings in Alzheimer's disease. Alzheimer's Research & Therapy, 12(1), 22. 10.1186/s13195-020-00587-5 PMC7057642 32131891

[brb32499-bib-0002] Ashburner, J. , & Friston, K. J. (2005). Unified segmentation. Neuroimage, 26(3), 839–851. 10.1016/j.neuroimage.2005.02.018 15955494

[brb32499-bib-0003] Battle, M. R. , Pillay, L. C. , Lowe, V. J. , Knopman, D. , Kemp, B. , Rowe, C. C. , Doré, V. , Villemagne, V. L. , & Buckley, C. J. (2018). Centiloid scaling for quantification of brain amyloid with [^18^F]flutemetamol using multiple processing methods. EJNMMI Research, 8(1), 107. 10.1186/s13550-018-0456-7 30519791 PMC6281542

[brb32499-bib-0004] Cho, H. , Choi, J. Y. , Hwang, M. S. , Kim, Y. J. , Lee, H. M. , Lee, H. S. , Lee, J. H. , Ryu, Y. H. , Lee, M. S. , & Lyoo, C. H. (2016). In vivo cortical spreading pattern of tau and amyloid in the Alzheimer disease spectrum. Annals of Neurology, 80(2), 247–258. 10.1002/ana.24711 27323247

[brb32499-bib-0005] Collij, L. E. , Konijnenberg, E. , Reimand, J. , Kate, M. T. , Braber, A. D. , Lopes Alves, I. , Zwan, M. , Yaqub, M. , van Assema, D. , Wink, A. M. , Lammertsma, A. A. , Scheltens, P. , Visser, P. J. , Barkhof, F. , & van Berckel, B. (2019). Assessing amyloid pathology in cognitively normal subjects using ^18^F‐Flutemetamol PET: Comparing visual reads and quantitative methods. Journal of Nuclear Medicine, 60(4), 541–547. 10.2967/jnumed.118.211532 30315145 PMC6448465

[brb32499-bib-0006] Hanseeuw, B. J. , Betensky, R. A. , Mormino, E. C. , Schultz, A. P. , Sepulcre, J. , Becker, J. A. , Jacobs, H. , Buckley, R. F. , LaPoint, M. R. , Vannini, P. , Donovan, N. J. , Chhatwal, J. P. , Marshall, G. A. , Papp, K. V. , Amariglio, R. E. , Rentz, D. M. , Sperling, R. A. , Johnson, K. A. , Alzheimer's Disease Neuroimaging Initiative , & Harvard Aging Brain Study (2018). PET staging of amyloidosis using striatum. Alzheimer's & Dementia, 14(10), 1281–1292. 10.1016/j.jalz.2018.04.011 PMC6219621 29792874

[brb32499-bib-0007] Hosokawa, C. , Ishii, K. , Kimura, Y. , Hyodo, T. , Hosono, M. , Sakaguchi, K. , Usami, K. , Shimamoto, K. , Yamazoe, Y. , & Murakami, T. (2015). Performance of 11C‐Pittsburgh compound B PET binding potential images in the detection of amyloid deposits on equivocal static images. Journal of Nuclear Medicine, 56(12), 1910–1915. 10.2967/jnumed.115.156414 26359262

[brb32499-bib-0008] Huang, K. L. , Lin, K. J. , Hsiao, I. T. , Kuo, H. C. , Hsu, W. C. , Chuang, W. L. , Kung, M. P. , Wey, S. P. , Hsieh, C. J. , Wai, Y. Y. , Yen, T. C. , & Huang, C. C. (2013). Regional amyloid deposition in amnestic mild cognitive impairment and Alzheimer's disease evaluated by [^18^F]AV‐45 positron emission tomography in Chinese population. Plos One, 8(3), e58974. 10.1371/journal.pone.0058974 23516589 PMC3597555

[brb32499-bib-0009] Klunk, W. E. , Koeppe, R. A. , Price, J. C. , Benzinger, T. L. , Devous, M. D. , Sr Jagust, W. J. , Johnson, K. A. , Mathis, C. A. , Minhas, D. , Pontecorvo, M. J. , Rowe, C. C. , Skovronsky, D. M. , & Mintun, M. A. (2015). The Centiloid Project: Standardizing quantitative amyloid plaque estimation by PET. Alzheimer's & Dementia, 11(1), 1–15.e154. 10.1016/j.jalz.2014.07.003 PMC4300247 25443857

[brb32499-bib-0010] La Joie, R. , Ayakta, N. , Seeley, W. W. , Borys, E. , Boxer, A. L. , DeCarli, C. , Doré, V. , Grinberg, L. T. , Huang, E. , Hwang, J. H. , Ikonomovic, M. D. , Jack, C. , Jr, J. , W, J. , Jin, L. W. , Klunk, W. E. , Kofler, J. , Lesman‐Segev, O. H. , Lockhart, S. N. , … Rabinovici, G. D. (2019). Multisite study of the relationships between antemortem [^11^C]PIB‐PET Centiloid values and postmortem measures of Alzheimer's disease neuropathology. Alzheimer's & Dementia: The Journal of the Alzheimer's Association, 15(2), 205–216. 10.1016/j.jalz.2018.09.001 PMC6368897 30347188

[brb32499-bib-0011] Matsuda, H. , Ito, K. , Ishii, K. , Shimosegawa, E. , Okazawa, H. , Mishina, M. , Mizumura, S. , Ishii, K. , Okita, K. , Shigemoto, Y. , Kato, T. , Takenaka, A. , Kaida, H. , Hanaoka, K. , Matsunaga, K. , Hatazawa, J. , Ikawa, M. , Tsujikawa, T. , Morooka, M. , … Sato, N. (2021). Quantitative evaluation of ^18^F‐Flutemetamol PET in patients with cognitive impairment and suspected Alzheimer's disease: A multicenter study. Frontiers in Neurology, 11, 578753. 10.3389/fneur.2020.578753 33519667 PMC7838486

[brb32499-bib-0012] Mattsson, N. , Insel, P. S. , Donohue, M. , Landau, S. , Jagust, W. J. , Shaw, L. M. , Trojanowski, J. Q. , Zetterberg, H. , Blennow, K. , Weiner, M. W. , & Alzheimer's Disease Neuroimaging Initiative (2015). Independent information from cerebrospinal fluid amyloid‐β and florbetapir imaging in Alzheimer's disease. Brain, 138(Pt 3), 772–783. 10.1093/brain/awu367 25541191 PMC4339769

[brb32499-bib-0013] Navitsky, M. , Joshi, A. D. , Kennedy, I. , Klunk, W. E. , Rowe, C. C. , Wong, D. F. , Pontecorvo, M. J. , Mintun, M. A. , & Devous, M. D. (2018). Standardization of amyloid quantitation with florbetapir standardized uptake value ratios to the Centiloid scale. Alzheimer's & Dementia, 14(12), 1565–1571. 10.1016/j.jalz.2018.06.1353 30006100

[brb32499-bib-0014] Palmqvist, S. , Schöll, M. , Strandberg, O. , Mattsson, N. , Stomrud, E. , Zetterberg, H. , Blennow, K. , Landau, S. , Jagust, W. , & Hansson, O. (2017). Earliest accumulation of β‐amyloid occurs within the default‐mode network and concurrently affects brain connectivity. Nature Communications, 8(1), 1214. 10.1038/s41467-017-01150-x PMC5663717 29089479

[brb32499-bib-0015] Rowe, C. C. , Jones, G. , Doré, V. , Pejoska, S. , Margison, L. , Mulligan, R. S. , Chan, J. G. , Young, K. , & Villemagne, V. L. (2016). Standardized expression of ^18^F‐NAV4694 and ^11^C‐PiB β‐amyloid PET results with the Centiloid scale. Journal of Nuclear Medicine, 57(8), 1233–1237. 10.2967/jnumed.115.171595 26912446

[brb32499-bib-0016] Rowe, C. C. , Doré, V. , Jones, G. , Baxendale, D. , Mulligan, R. S. , Bullich, S. , Stephens, A. W. , De Santi, S. , Masters, C. L. , Dinkelborg, L. , & Villemagne, V. L. (2017). ^18^F‐Florbetaben PET beta‐amyloid binding expressed in Centiloids. European Journal of Nuclear Medicine and Molecular Imaging, 44(12), 2053–2059. 10.1007/s00259-017-3749-6 28643043 PMC5656696

[brb32499-bib-0017] Villeneuve, S. , Rabinovici, G. D. , Cohn‐Sheehy, B. I. , Madison, C. , Ayakta, N. , Ghosh, P. M. , La Joie, R. , Arthur‐Bentil, S. K. , Vogel, J. W. , Marks, S. M. , Lehmann, M. , Rosen, H. J. , Reed, B. , Olichney, J. , Boxer, A. L. , Miller, B. L. , Borys, E. , Jin, L. W. , Huang, E. J. , … Jagust, W. (2015). Existing Pittsburgh Compound‐B positron emission tomography thresholds are too high: Statistical and pathological evaluation. Brain, 138(Pt 7), 2020–2033. 10.1093/brain/awv112 25953778 PMC4806716

